# Application of FTIR-PAS in Rapid Assessment of Rice Quality under Climate Change Conditions

**DOI:** 10.3390/foods10010159

**Published:** 2021-01-14

**Authors:** Lianlian Wei, Fei Ma, Changwen Du

**Affiliations:** 1 The State Key Laboratory of Soil and Sustainable Agriculture, Institute of Soil Science, Chinese Academy of Sciences, Nanjing 210008, China; llwei@issas.ac.cn (L.W.); fma@issas.ac.cn (F.M.); 2 College of Advanced Agricultural Sciences, University of Chinese Academy of Sciences, Beijing 100049, China

**Keywords:** Fourier transform infrared photoacoustic spectroscopy, climate change, rice quality, principal component analysis

## Abstract

Fourier transform infrared photoacoustic spectroscopy (FTIR-PAS), versus attenuated total reflectance spectroscopy (FTIR-ATR) and diffuse reflectance spectroscopy (DRIFT), was firstly applied in quick assessment of rice quality in response to rising CO_2_/temperature instead of conventional time-consuming chemical methods. The influences of elevated CO_2_ and higher temperature were identified using FTIR-PAS spectra by principal component analysis (PCA). Variations in the rice functional groups are crucial indicators for rice identification, and the ratio of the intensities of two selected spectral bands was used for correlation analysis with starch, protein, and lipid content, and the ratios all showed a positive linear correlation (*R*^2^ = 0.9103, *R*^2^ = 0.9580, and *R*^2^ = 0.9246, respectively). Subsequently, changes in nutritional components under future environmental conditions that encompass higher CO_2_ and temperature were evaluated, which demonstrated the potential of FTIR-PAS to detect the responses of rice to climate change, providing a valuable technique for agricultural production and food security.

## 1. Introduction

Temperature and CO_2_ concentration are major environmental variables influencing crops’ growth and are expected to change rapidly in the future, which will impact food security [1]. Rice (*Oryza sativa* L.) is one of the most important crops in the world and provides a daily source of food for over 2 billion people [2], with approximately 600 million people relying on rice for more than 50% of their daily calories [3]. Nutrition of rice intake is of obvious importance for promotion and maintenance of human health, and any changes associated with nutritional availability for rice might have global health consequences [4,5].

For the time being, the majority of nutritional information for rice has been obtained primarily from single climate variables; i.e., either CO_2_ or temperature. Results from a meta-analysis indicated that rice grain protein content was reduced by CO_2_ concentration enrichment [6]. There were also considerable studies indicating that future CO_2_ concentration will reduce protein [3,7,8], while the amylose content responds more variably to elevated CO_2_ [8,9,10]. Temperature effects on rice quality were more disparate, although there were evidences indicating that temperature may also negatively influence rice quality [11,12]. For instance, rice grains produced under rising temperature exhibited lower amylose content [13], and the protein content was increased or not affected under elevated temperature 2–4 °C [7,9,11]; however, the interactions of temperature and CO_2_ concentration remain unclear, and it is necessary to investigate the interactions regarding the main nutritional items including starch, protein and lipids.

Frequently used methods for assessing rice nutrition have been based on chemical determination. Conventional methods for determining the starch content of rice grains include the amyloglucosidase-α-amylase method [14]. Relatively common traditional methods for analyzing protein content include the Dumas method, Kjeldahl methods, and AOAC (Association of Official Analytical Chemists) official method 979.09 [15,16]. Lipid content is quantified by the traditional Soxhlet extraction method [17] and the acid hydrolysis method [18]. The analysis process of these chemical methods is time-consuming and laborious because of sample pretreatment, destructive owing to digestion procedures, and even dangerous owing to the use of corrosive and potentially toxic reagents. Thus, a fast and nondestructive method to determine the grain nutrition of rice is in high demand.

**Figure 1:**
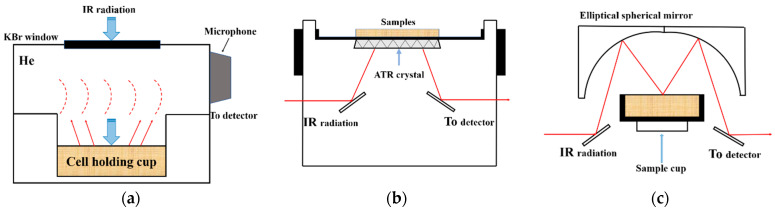
Schematic diagram of light path of (**a**) Fourier transform infrared photoacoustic spectroscopy (FTIR-PAS), (**b**) Fourier transform infrared attenuated total reflectance spectroscopy (FTIR-ATR), and (**c**) diffuse reflectance Fourier transform infrared spectroscopy (DRIFTS).

For this purpose, much attention has been focused on near-infrared reflectance spectroscopy (NIRS) as an alternative option to traditional means. A number of studies have shown that NIRS has the potential to be used as a quick procedure for predicting the quality properties involving amylose content, protein content, and other grain quality traits [19,20,21,22]. However, weak absorption of overtone and combination, broad bands and lack of characteristics are typical of NIRS, thus making NIRS calibration models largely dependent on chemometric methods. Instead, mid-infrared spectroscopy (MIRS) was found to have more potential to characterize various samples, since it displays stronger basic frequency absorption and more well-resolved spectral features associated with the sample component. Also, photoacoustic spectroscopy-FTIR (FTIR-PAS), attenuated total reflectance-FTIR spectroscopy (FTIR-ATR), and diffuse reflectance FTIR spectroscopy (DRIFT) are commonly used MIRS for rapid analysis [23,24,25,26,27]. Recent progress in microphone sensitivity has dramatically increased the performance of FTIR-PAS. The advantage of FTIR-PAS is that the shape of the photoacoustic spectrum is independent of the sample’s morphology [28]. Spectra of FTIR-ATR are obtained from the absorption of an evanescent wave, which is transmitted through an internal reflection element with a high refractive index and penetrates the sample [29,30]. Direct contact between the sample and the ATR crystal is required, which can be achieved easily using liquids, pastes and powders [28]. Diffuse reflectance spectroscopy is based on the detection of electromagnetic radiation reflected at a characteristic wavelength and does not require direct contact between the sensor and the samples. The energy that penetrates one or more particles is reflected in all directions [26,28,29]. Schematic diagram of light path of FTIR-PAS, FTIR-ATR, and DRIFTS are shown in Figure 1.

In this paper, FTIR-PAS was firstly applied to characterize and identify rice grain samples from temperature/free air CO_2_ enrichment (T-FACE) platform, and subsequently, the objective of this study was to rapidly analyze the selected nutritional properties (starch, protein, and lipid content) in milled rice grain of one variety in response to climate change using FTIR-PAS spectra.

## 2. Materials and Methods

### 2.1. Experimental Site

The experiment was carried out in Yangzhou, Jiangsu Province, China (32°355″ N, 119°420″ E). This region has a typical subtropical monsoon climate, with an average annual temperature of 14.9 °C and annual precipitation of 980 mm. The soil is classified as a Shajiang Aquic Cambosol with a sandy loam texture. The details of the soil properties are as follows: sand (0.02–2 mm) 578.4 g kg^−1^, silt (0.002–0.02 mm) 285.1 g kg^−1^, clay (<0.002 mm) 136.5 g kg^−1^, bulk density 1.16 g cm^−3^, soil organic C 18.4 g kg^−1^, total N 1.45 g kg^−1^, total P 0.63 g kg^−1^, total K 14.0 g kg^−1^, available P 10.1 mg kg^−1^, available K 70.5 mg kg^−1^, and pH 7.2.

### 2.2. T-FACE Facility

The method for controlling CO_2_ and temperature in situ has been described in detail by Cai et al. [31] and Wang et al. [32,33]. The temperature and free-air CO_2_ enrichment (T-FACE) system is characterized by six rings located within a paddy field with a similar soil and cultivation history. For the higher CO_2_ treatment, three octagonal plots were randomly allocated to the elevated CO_2_ (eC) treatments and the other three to the ambient (aC) conditions. To avoid CO_2_ contamination, each plot was separated by at least 90 m. The FACE plots were equipped with emission tubes around the perimeter at heights of 50–60 cm above the canopy, which released CO_2_ in the day time throughout the growing season to maintain a CO_2_ concentration at approximately 200 µmol mol^−1^ above ambient levels. To maintain the elevated CO_2_ concentration, the FACE plots were controlled by computer systems that monitored the ambient CO_2_ concentration variation, wind direction, wind speed, and canopy height during daylight hours. The plants received additional CO_2_ from 29 June to 25 October during the day time. The average concentrations of ambient and elevated CO_2_ were 384.7 ± 4.5 µmol mol^−1^ and 574.0 ± 5.4 µmol mol^−1^, respectively. No attempt was made to control CO_2_ in the ambient plots. Temperature treatment was imposed for each plot as a split-plot. In brief, we established a plot for elevated temperature (eT) by encircling a 2.7 m × 5.25 m area with copper pipes. In the eT plots, temperature was elevated by approximately 1.5 °C at the canopy height through coordination between a temperature sensor (SI-109, Campbell, CA, USA) and a computer feedback system that controlled the speed of the running hot water in the copper pipes. Temperature rising lasted from 29 June to 25 October, daily from 09:00 h to 18:00 h. The average increment of elevated temperature was 1.4 ± 0.3 °C higher than the ambient. Temperatures were not elevated in the other half of each plot. Overall, the study had four treatments: (1) aCaT (ambient treatment), (2) aCeT (ambient CO_2_ concentration and increased temperature), (3) eCaT (increased CO_2_ concentration and ambient temperature), and 4) eCeT (combination of elevated CO_2_ and increased temperature). The CO_2_ and temperature metrics were chosen based on IPCC (Intergovernmental Panel on Climate Change) RCP 4.5 (Representative Concentrative Pathway) scenario predictions of ca 500–700 mg L^−1^ CO_2_ and 1.1–2.6 °C temperature increases by century’s end [34].

### 2.3. Crop Cultivation and Experimental Treatment

Nanjing 9108 (NJ9108), a commercial japonica rice cultivar authorized by the China Ministry of Agriculture, was used. NJ9108 is recognized as a high-yield and high-grain quality cultivar in Jiangsu Province, where the experiment was conducted. Jiangsu Province is the fifth most productive rice province in China [35].

Rice seedlings were selected and manually transplanted at a density of two seedlings per hill. The spacing of the hills was 16.7 cm × 25 cm (equivalent to 24 hills m^−2^). All plots received equal amounts of phosphorus (P) and potassium (K) as the basal dressing 1 day before transplanting, when both PK compound chemical fertilizers were applied at a rate of 9 g m^−2^. Nitrogen (N) was supplied as urea (N = 46%) and compound chemical fertilizer (N:P_2_O_5_:K_2_O = 15:15:15) at a rate of 22.5 g N m^−2^. Of the total N, 40% was applied as the basal dressing 1 day before transplanting, 30% was top-dressed at the early tillering stage, and 30% was top-dressed at the panicle initiation stage.

At maturity, aboveground plant parts were harvested by hand with a sickle from the whole treatment area. Subsequently, grains were husked by rice huller (JLG-II, Grain Reserves Corporation, Beijing, China) to produce brown rice. To ensure consistent removal efficiency, equal weight brown rice was taken and then removed from the bran layer by a rice polisher (JNM-III, Grain Reserves Corporation, Beijing, China) to obtain white rice. Then, a ball mill (MM400, Retsch, Germany) was used to grind white rice separately into powder samples that could pass through a 100-mesh (0.15 mm) sieve. After drying (70 °C, 6 h), the sample was placed in a desiccator and cooled to room temperature for further analyses. All samples were analyzed for three analytical replicates. The moisture content of the samples was displayed in the Supplementary Materials Table S1.

### 2.4. Spectra Recording

#### 2.4.1. FTIR-PAS Spectra Acquisition

Rice grain sample spectra were collected using a Nicolet 6700 FTIR spectrometer (Thermo Scientific, Waltham, MA, USA) coupled with a Model 300 photoacoustic cell (MTEC Photoacoustics, Inc., Ames, USA). The sample was placed in a cell holding cup (diameter of 10 mm and height of 5 mm), after which the cell was purged with dry helium (10 mL min^−1^) for 20 s to minimize the interference caused by CO_2_ and H_2_O. Thirty-two successive scans were performed in the range of 400–4000 cm^−1^ with a resolution of 4 cm^−1^ and a mirror velocity of 0.32 cm s^−1^. Carbon black was used as the background for intensity normalization of the spectra.

#### 2.4.2. Attenuated Total Reflectance Fourier-Transform Infrared Spectroscopy

The FTIR-ATR spectra were scanned on a handheld TruDefender Fourier transform spectrometer (Thermo Fisher Scientific, Waltham, MA, USA). Spectra were acquired in the range 4000–650 cm^−1^ with a spectral resolution of 4 cm^−1^. Diamond was used as the ATR-reflecting element for improved contact, and a blank reference was scanned before each sample was scanned. The background was subtracted from each scan to correct for atmospheric and instrumental noise.

#### 2.4.3. Diffuse Reflectance Fourier-Transform Infrared Spectroscopy

Diffuse reflectance Fourier transform mid-infrared spectra were collected on a Nicolet 6700 spectrophotometer (Thermo Fisher Scientific, Waltham, MA, USA) fitted with a diffuse reflectance accessory (PN 044–10XX 300, PIKE Technologies, Fitchburg, WI, USA). Spectra were collected as an average of 32 scans at a resolution of 4 cm^−1^ within the range from 4000–400 cm^−1^ [18,19,20,25]. A gold reference was used as a background at the beginning of each sample scan. The background was subtracted from each scan to correct for atmospheric and instrumental noise.

### 2.5. Chemical Methods

Ethanol (10 mL) was added to a 100 mg sample of rice flour and kept in a water bath at 80 °C for 30 min. The tube was then centrifuged at 2000 rpm for 20 min after cooling and repeated three times. The residue in the centrifuge tube was dried at 80 °C for starch extraction. Then, 2 mL of distilled water was added to the tube, which was shaken in a boiling water bath for 15 min, and 2 mL of 9.36 HClO_4_ was added after cooling. The solution was shaken for 15 min, made up to approximately 10 mL, and centrifuged at 2000 rpm for 20 min. The supernatant was collected and 2 mL of 4.68 HClO_4_ was added to the residue. The extraction was repeated as described above, then the supernatant was added, and the volume was made up to 50 mL with distilled water. The starch content was measured according to the method of Pucher et al. [36]. The N content was determined using the Kjeldahl method and multiplied by 5.95 to obtain the protein content. The lipids were extracted from the rice using a FOSS Soxtec 2050 [17]. In brief, 5 g of rice was weighed into cellulose thimbles (Foss North America, Eden Prairie, MN, USA). Then, the thimbles and kernels were pre-dried for 1 h in a 100 °C oven. Subsequently, the lipids were extracted from the sample using 70 mL of petroleum ether (boiling point 35–60 °C; VWR, Suwanee, GA). Samples were boiled in the solvent for 20 min over a 135 °C hot plate, rinsed with petroleum ether condensate for 30 min, and dried for 5 min. After the extraction cycle, the extraction cups were removed from the Soxtec unit and placed in an oven maintained at 100 °C for 30 min to evaporate the solvent. The extraction cups were placed in a desiccator at room temperature for approximately 30 min to cool before weighing. The difference between the mass of the cups containing the extracted lipid and the original empty cup mass was calculated to obtain the mass of the extracted lipid. Lipid content was expressed as the mass percentage of the extracted lipid mass to the original one.

### 2.6. Statistical Analysis

Both FTIR-PAS and DRIFT data were first truncated to span a smaller wavelength range (4000–650 cm^−1^) identical to the FTIR-ATR data on account of FTIR-ATR spectra and were recorded on an instrument different from that used to record FTIR-PAS and DRIFT spectra. The spectra recorded using the FTIR-PAS, FTIR-ATR, and DRIFT techniques for all the samples were smoothed using the Savitzky–Golay filter and subsequently averaged. The scanning depth of the sample can be calculated as follows (Equation (1)):

(1) $$u=\sqrt{\frac{D}{\pi vr}}$$

where *u* is the thermal diffusion length (μm), *D* is the thermal diffusivity, *v* is the moving mirror velocity, and *r* is the wavenumber. *D* is approximately 0.01 × 10^−5^ m^2^ s^−1^.

The FTIR-PAS spectra were smoothed using the Savitzky–Golay filter and subsequently averaged and normalized. PCA was performed as exploratory data analysis in order to obtain an overview of the variation among samples and to identify clusters and outliers. This step is applied to reduce the spectral information into principal components (PCs), which are a linear combination of the variables in the spectral data and contain most of the relevant information. The spectral pre-processing, PCA, and statistical analysis were implemented in MATLAB R2016a (The Math Works, Natick, MA, USA). Curve fitting was conducted using a Peakfit v.4.12 (SeaSolve Software Inc. San Jose, CA, USA). Analysis of variance (ANOVA) was performed using SPSS v.20.0 for Windows (SPSS Inc., Chicago, IL, USA).

## 3. Results and Discussion

### 3.1. Spectral Characterization

**Figure 2:**
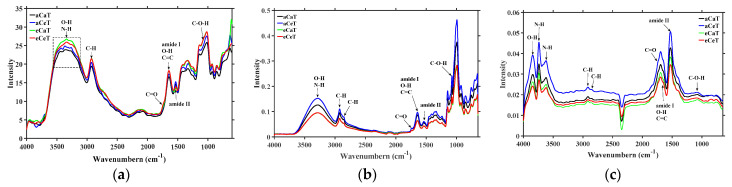
Averaged spectra of (**a**) Fourier transform infrared photoacoustic spectroscopy (FTIR-PAS), (**b**) Fourier transform infrared attenuated total reflectance spectroscopy (FTIR-ATR), and (**c**) Fourier transform infrared diffuse reflectance spectroscopy (DRIFTS) of rice samples under different treatments. Note: aCaT, aCeT, eCaT, and eCeT refer to ambient conditions, increased temperature, elevated CO_2_, and the combination of elevated CO_2_ and increased temperature, respectively.

For the same technique, the spectra of rice samples from different treatments displayed very similar shapes and peaks positions but differed in intensity across the spectral region (Figure 2), which indicated that the rice samples had the same composition but varied in amounts. FTIR-PAS and FTIR-ATR provided similar spectra and contained more features than the spectra recorded by DRIFT.

A broad and strong peak attributed to O-H stretching vibration absorption [37,38] overlapping with N-H stretching vibration (amide I and amide II) [39] was observed from FTIR-PAS and FTIR-ATR in the region 3800–3000 cm^−1^, whereas DRIFT showed a separate and shift of the peaks toward higher wavenumbers (at 3843 cm^−1^, 3735 cm^−1^ and 3618 cm^−1^). The peak related to the asymmetric stretching vibration of methylene at 2920 cm^−1^ [40] was clearly visible in all techniques, while the peak related to the symmetric stretching vibration of aliphatic methylene at 2850 cm^−1^ [26] was just present in FTIR-ATR and DRIFT. Diffuse reflectance-FTIR was more sensitive to carbon dioxide in the environment than FTIR-PAS and FTIR-ATR with a strong CO_2_ absorption peak around 2350 cm^−1^. The spectral region between 1800 and 900 cm^−1^ was called the fingerprint region because of the unique patterns’ characteristic of a given sample [41]. More resolved important characteristic peaks in the fingerprint region were clearly visible in the recorded FTIR-PAS and FTIR-ATR spectra, but FTIR-ATR spectra were less detailed and nosier than the FTIR-PAS in the region 1200–1500 cm^-1^. Furthermore, DRIFT seemed to be missing characteristic peaks in the region 1300–900 cm^−1^, which includes information related to the C-O stretching/C-C stretching/C-O-C stretching (900–1200 cm^−1^) in starch and lipid, and C-N stretching/N-H bending (1240 cm^−1^) in protein [37,38,42,43].

Table 1 shows the functional groups and modes of vibration according to the most observed prominent absorption bands. The recorded mid-infrared spectra of rice revealed a better performance overall of FTIR-PAS and FTIR-ATR than of DRIFT spectra.

**Figure 3:**
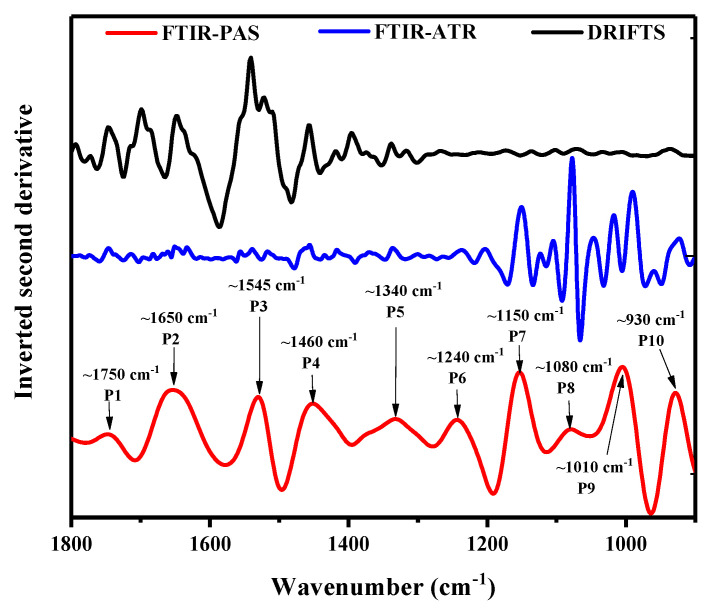
Inverted second-derivative curves of Fourier transform infrared photoacoustic spectroscopy (FTIR-PAS), Fourier transform infrared attenuated total reflectance spectroscopy (FTIR-ATR), and Fourier transform infrared diffuse reflectance spectroscopy (DRIFTS) spectra in the range of 900–1800 cm^−^^1^.

In order to acquire precise characteristic peaks’ number and position, second-derivative analysis was used, and the second-derivative spectra in the fingerprint region are shown in Figure 3. Second-derivative spectra of FTIR-ATR and DRIFT were incapable of extracting more information than FTIR-PAS spectra, especially in the 1800–1200 cm^−1^ and 1500–900 cm^−1^ regions respectively, which were dominated by noise.

The CV (coefficient of variation) of characteristic peaks’ intensity within group and between groups are exhibited in Table 2. FTIR-PAS had a smaller coefficient of variation within the group, followed by FTIR-ATR and DRIFT. This manifested that FTIR-PAS spectroscopy was characterized by high reproducibility. The main advantage of FTIR-ATR was small optical contact, and the main drawback of DRIFT was strongly dependent on particle size and packing density. All these issues might result in the problem in the method’s reproducibility, and FTIR-PAS seemed to be the most appropriate technique for the rice samples [44].

### 3.2. Principal Component Analysis (PCA)

**Figure 4:**
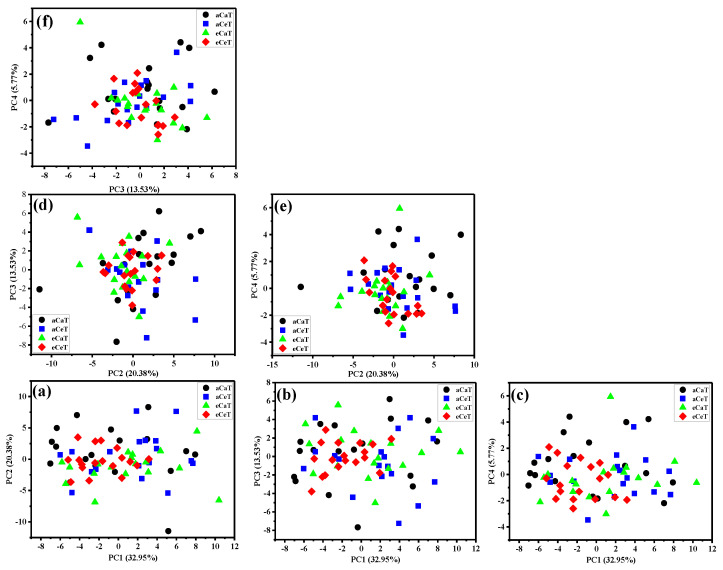
Principal component distributions of rice based on FTIR-PAS spectra (600–4000 cm^−1^). (**a**): PC1-PC2 scatterplot; (**b**): PC1-PC3 scatterplot; (**c**): PC1-PC4 scatterplot; (**d**): PC2-PC3 scatter-plot; (**e**): PC2-PC4 scatterplot; (**f**): PC3-PC4 scatterplot; Note: aCaT, aCeT, eCaT, and eCeT refer to ambient conditions, increased temperature, elevated CO_2_, and the combination of elevated CO_2_ and increased temperature, respectively.

Figure 4 shows the scatter plots associated with PC1, PC2, PC3, and PC4 at full-spectrum wavenumber ranges. The first four principal components accounted for 32.95%, 20.38%, 13.53%, and 5.77% of the total variance, respectively. The score plots exhibited an irregular pattern across all of the samples, indicating that some treatments were difficult to distinguish. The poor performance of PCA based on full wavelength may be attributed to some useless and irrelevant information included in the full spectrum [25,45].

**Figure 5:**
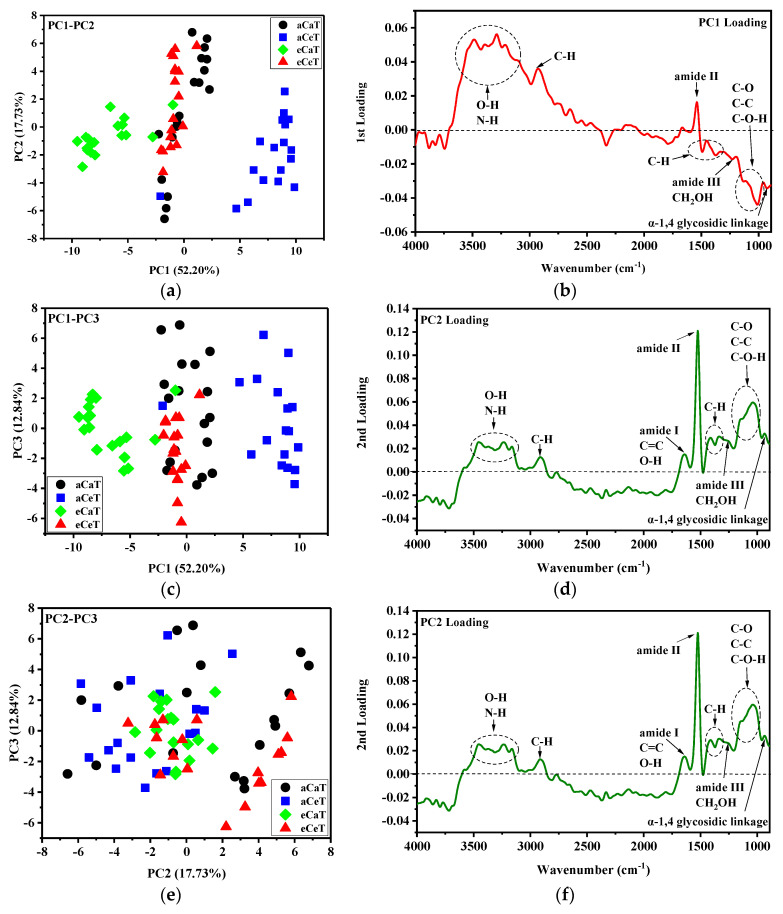
Principal component score distributions (**a**–**c**) and principal component loading plots (**d**–**f**) of the first three principal components from the FTIR-PAS spectra of the rice in the range of 900–4000 cm^−1^. Note: aCaT, aCeT, eCaT, and eCeT refer to ambient conditions, increased temperature, elevated CO_2_, and the combination of elevated CO_2_ and increased temperature, respectively.

Figure 5 shows the score plots of the first three principal components in the wavenumber ranges of 900–4000 cm^−1^. The first three principal components accounted for 52.50%, 17.73%, and 12.84% of the total variance, respectively. Cumulatively, the first three PCA scores accounted for 82.77% of the total variance. The fourth and other remaining components contributed less than 5% of the residual variance. Therefore, the first three PCA scores (PC1, PC2, and PC3) were selected for further analysis because they contained substantial information about the spectra of the rice samples. Different treatments were effortlessly distinguishable from the scatterplots of PC1 versus PC2 and PC1 versus PC3, suggesting that significant differences existed among the rice samples under different treatments. The influence of elevated CO_2_ can be clearly observed through the distinction of aCaT from eCaT and of aCeT from eCeT. The same effects were detected for the impacts of elevated temperature: The aCaT and aCeT samples showed discrepancies, and the eCaT were clearly distinguishable from eCeT. Beyond that, however, the majority of PCA score plots for the aCaT and eCeT samples overlapped to a certain extent.

These results illustrate that the two-factor interaction effects between elevated CO_2_ and elevated temperature can neutralize each other. According to the loading plots (Figure 5d–f), high absolute values of PC1, PC2, and PC3 loadings were observed at the wavenumbers of the amide I/C=C/O-H (≈1650 cm^−1^), amide II (≈1545 cm^−1^), C-H (≈1340 cm^−1^, 1460 cm^−1^), amide III/CH_2_OH (≈1240 cm^−1^), C-O/C-C (≈1150 cm^−1^), C-O-H (≈1080 cm^−1^), C-O-C (≈1010 cm^−1^), and α-1,4 glycosidic linkage (≈930 cm^−1^), which might contribute to the diversity. The dispersion might show individual differences in the rice samples under different treatments.

### 3.3. Curve Fitting through Deconvolution

**Figure 6:**
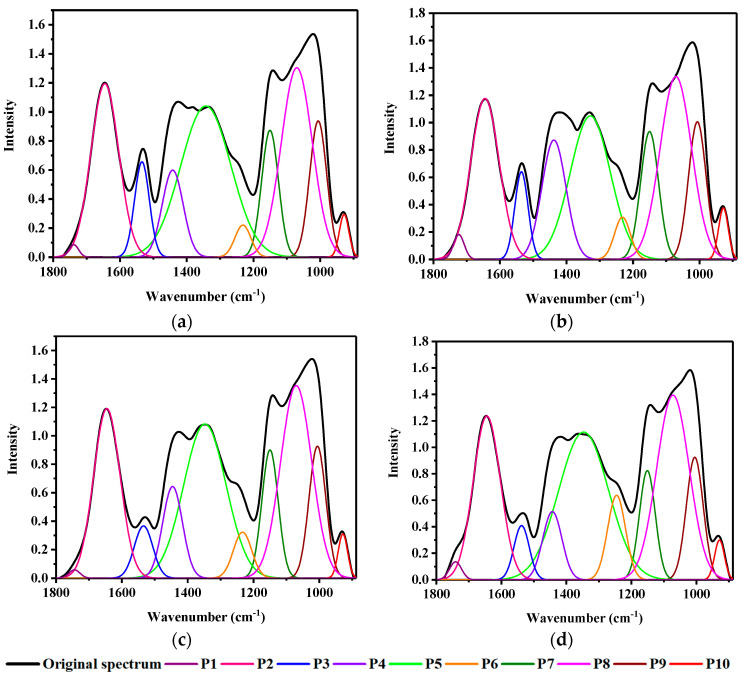
Deconvolution curve-fitting with four different treatments. (**a**) aCaT: ambient CO_2_ and temperature; (**b**) aCeT: ambient CO_2_ and elevated temperature; (**c**) eCaT: elevated CO_2_ and ambient temperature; (**d**) eCeT: elevated CO_2_ and temperature.

In the region 1800–900 cm^−1^, center positions for each sub-band in curve-fitting were determined by second-derivative analysis, and the shapes of the underlying bands were chosen using a Gaussian algorithm (Figure 3). The spectrum in this region was divided into 10 isolated peaks (Figure 6). The correlation coefficient (*R*^2^) between the raw spectrum and fitted spectrum was above 0.95, and the standard error was less than 0.1. The deconvolution curve fitting for the different treatments is shown in Figure 6.

### 3.4. Detection of Rice Nutritional Parameters

In the spectra, to avoid interference from the equipment and the environment while measuring differences in the absolute values of the peak intensities [45,46], the ratio of two selected spectral bands was calculated. Two peaks at approximately 1080 cm^−1^ and 1650 cm^−1^ were attributed to C-O-H stretching vibration and O-H bending vibration (water adsorbed in the amorphous regions) from starch, respectively, which could be assessed for relative starch content [47]. Amide has been shown to be the main chemical form for nitrogen storage in plants. Therefore, the peak at approximately 1545 cm^−1^ was assigned to C=O stretching (amide I), and 1650 cm^−1^ was assigned to amide II, which corresponded to the C-N stretching and N-H bending, respectively, and were selected to detect relative protein content [45,46]. Relative lipid content was determined by the two peaks at approximately 1740 cm^−1^, which was assigned to C=O stretching vibration, and 1650 cm^−1^ stretching vibration, which was attributed to C=C stretching vibration [47].

**Figure 7:**
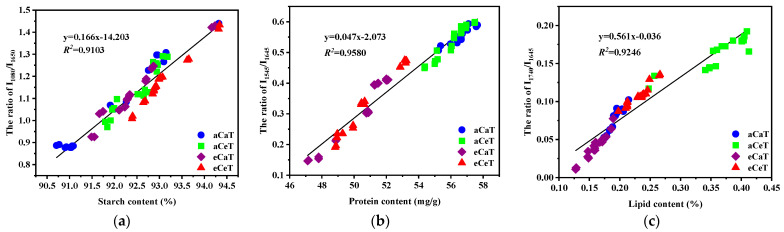
Linear regression between (**a**) starch content and the ratio of I_1080_/I_1650_; (**b**) protein content and the ratio of I_1545_/I_1650_; and (**c**) lipid content and the ratio of I_1750_/I_1650_. Note: aCaT, aCeT, eCaT, and eCeT refer to ambient conditions, increased temperature, elevated CO_2_, and the combination of elevated CO_2_ and increased temperature, respectively. The linear functions and the square of the correlation coefficient (*R*^2^) are shown in each plot.

**Figure 8:**
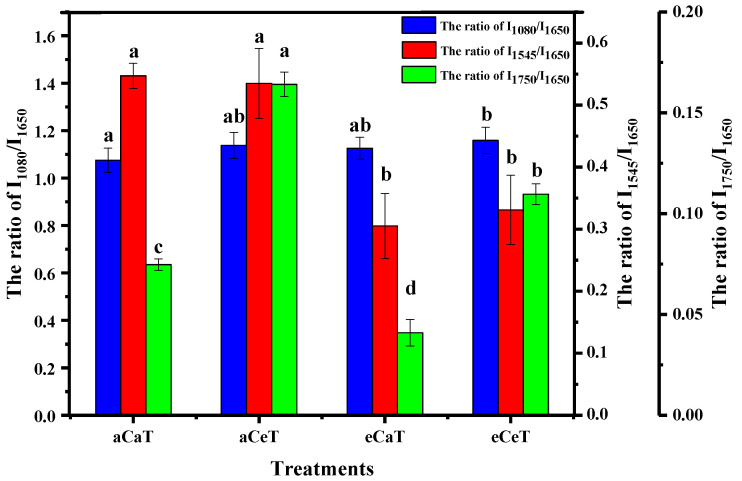
The photoacoustic intensity ratio of I_1080_/I_1650_, I_1545_/I_1650_, and I_1750_/I_1650_. Note: aCaT, aCeT, eCaT, and eCeT refer to ambient conditions, increased temperature, elevated CO_2_, and the combination of elevated CO_2_ and increased temperature, respectively. Bars represent the standard error of the means. In each column, the values followed by different letters indicate a significant difference at *p* < 0.05 according to the Duncan test.

The starch content of the rice showed a positive linear correlation (*R*^2^ = 0.9103) with the intensity ratio of I_1080_/I_1650_ (Figure 7a). The ratio of aCaT showed a minimal value of 1.074; however, the same ratio reached 1.138, 1.126, and 1.158 when the aCeT, eCaT, and eCeT treatments were used, respectively (Figure 8). A significant increase was only observed between aCaT and eCeT, indicating that there was no significant increase from either CO_2_ or temperature, and a significant increase was observed for concomitant increases in both variables relative to the control. Figure 7b shows that the correlation between the ratio of I_1545_/I_1650_ and protein content was relatively high, with a coefficient of 0.9580. From Figure 8, we can see that the ratios of all treatments displayed lower values relative to the control, and protein content was significantly decreased by elevated CO_2_ concentration. A high correlation (*R*^2^ = 0.9246) was observed between lipid content and I_1750_/I_1650_ (Figure 7c)_._ Warmer temperature and elevated CO_2_ resulted in opposing effects, increasing and decreasing lipid content, respectively. Owing to these opposing effects, a significant interaction was noted for both variables (Figure 8). Given the relative content of rice nutrition, any changes are consistent with previous reports measuring these parameters by chemical methods [6,7,8,48,49,50].

## 4. Conclusions

Three FTIR spectra (PAS, ATR, DRIFT) of the rice displayed similar shapes and dissimilar intensities across the spectral region in the different treatments of elevated CO_2_, increasing temperature and the combination of elevated CO_2_ and increased temperature. Comparing the three techniques, FTIR-PAS was the best technique for rice sample quantitative analysis due to being more detailed and less noisy. The FTIR-PAS spectra of the rice displayed similar shapes and dissimilar intensities across the spectral region in the different treatments of elevated CO_2_, increasing temperature, and the combination of elevated CO_2_ and increased temperature. Based on the principal component analysis, the effects of elevated CO_2_ and increasing temperature could be easily distinguished from FTIR-PAS spectra in the range of 900–4000 cm^−1^. The ratio of I_1080_/I_1650_ showed a positive linear correlation (*R*^2^ = 0.9103) with the starch content of rice, and a significant interaction effect was observed between elevated CO_2_ and temperature treatment. The correlation between the ratio of I_1545_/I_1650_ and protein content was relatively high, with a coefficient of 0.9580, and the protein content was significantly decreased by elevated CO_2_ concentration. A high correlation (*R*^2^ = 0.9246) was observed between lipid content and I_1750_/I_1650_, and the opposing effects of significantly increased lipid content from warmer temperatures and significantly reduced lipid content from elevated CO_2_ resulted in a significant interaction. Hence, nondestructive and rapid FTIR-PAS provides a novel and unique technique to characterize rice grains, which shows great potential for detecting how milled rice grains respond to future environmental conditions that encompass higher CO_2_ and temperature. This study also has its limitations: Regarding the method, the aspect of FTIR-PAS is relatively equipment-expensive compared with traditional biochemical methods in measuring carbohydrates, protein, and lipids; regarding material aspects, only one variety is evaluated using FTIR, and varied varieties grown in different environments are needed to validate the method in further explorations.

## Figures and Tables

**Table 1:** Assignment of the main bands of the FTIR spectra of the rice samples.

FTIR-PAS			FTIR-ATR		DRIFT	
Wavelength (cm^−1^)	Assignment	Profiling Depth (μm)	Wavelength (cm^−1^)	Assignment	Wavelength (cm^−1^)	Assignment
3600–3000	-O-H/-N-H	5.26–5.76	3600–3000	-O-H/-N-H	3843	-O-H
2920	-C-H	5.84	2920	-C-H	3735	-N-H
1750	-C=O	7.54	2850	-C-H	3618	-N-H
1650	amide I/-O-H/-C=C	7.77	1750	-C=O	2920	-C-H
1545	amide II	8.07	1650	amide I/-O-H/-C=C	2850	-C-H
1460	-C-O-O/-C-H	8.26	1530	amide II	1750	-C=O
1340	-C-H	8.62	1460	-C-O-O/-C-H	1650	amide I/-O-H/-C=C
1240	amideIII/-CH_2_OH	8.96	1340	-C-H	1530	amide II
1150	-C-O/-C-C	9.30	1240	amideIII/-CH_2_OH	1460	-C-O-O/-C-H
1080	-C-O-H	9.60	1150	-C-O/-C-C	1080	-C-O-H
1010	-C-O-C/-C-O	9.93	1080	-C-O-H	930	α-1,4 glycosidic linkage
930	α-1,4 glycosidic linkage	10.34	1010	-C-O-C/-C-O		
			930	α-1,4 glycosidic linkage		

FTIR-PAS: Fourier transform infrared photoacoustic spectroscopy; FTIR-ATR: Fourier transform infrared attenuated total reflectance spectroscopy; DRIFTS: Fourier transform infrared diffuse reflectance spectroscopy.

**Table 2:** CV of spectral intensity within group and between groups.

Coefficient of Variation		FTIR-PAS				FTIR-ATR				DRIFT			
		I_1080_	I_1545_	I_1650_	I_1750_	I_1080_	I_1530_	I_1650_	I_1750_	I_1080_	I_1530_	I_1650_	I_1750_
Within group	aCaT	0.032	0.058	0.041	0.057	0.075	0.112	0.035	0.143	0.111	0.218	0.157	0.140
	aCeT	0.051	0.067	0.043	0.066	0.094	0.121	0.034	0.037	0.089	0.437	0.245	0.065
	eCaT	0.067	0.099	0.091	0.051	0.125	0.154	0.050	0.310	0.127	0.555	0.349	0.152
	eCeT	0.057	0.100	0.113	0.061	0.150	0.167	0.161	0.152	0.097	0.337	0.307	0.113
Among groups		0.135	0.143	0.137	0.072	0.192	0.154	0.181	0.144	0.128	0.759	0.163	0.764

Note: CV, Coefficient of variation; aCaT, aCeT, eCaT, and eCeT refer to ambient conditions, increased temperature, elevated CO_2_, and the combination of elevated CO_2_ and increased temperature, respectively.

## Data Availability

Data available on request due to restrictions privacy.

## Supplementary Materials

The following are available online at https://www.mdpi.com/2304-8158/10/1/159/s1, Table S1: Statistics of main nutritional parameters for rice samples.
