# Supplementary material for: Application of FTIR-PAS in Rapid Assessment of Rice Quality under Climate Change Conditions
Source: Foods. 2021 Jan 14;10(1):159. doi: 10.3390/foods10010159 (PMC7828744; doi:10.3390/foods10010159)
Supplement: Supplementary file 1 [file foods-10-00159-s001.pdf]

Table S1. Statistics of selected nutritional parameters for rice samples.

Parameter	Treatment	Number	Mean	SD
Starch content (%)	aCaT	18	91.984 ^{b}	1.090
	aCeT	18	92.515 ^{ab}	0.466
	eCaT	18	92.437 ^{ab}	0.760
	eCeT	18	93.080 ^{a}	0.556
Protein content (mg g^{-1})	aCaT	18	56.464 ^{a}	0.688
	aCeT	18	56.042 ^{a}	0.984
	eCaT	18	50.255 ^{b}	1.786
	eCeT	18	50.816 ^{b}	1.755
Lipid content (%)	aCaT	18	0.191 ^{c}	0.012
	aCeT	18	0.365 ^{a}	0.048
	eCaT	18	0.160 ^{d}	0.017
	eCeT	18	0.231 ^{b}	0.019

Note: aCaT, aCeT, eCaT, and eCeT refer to ambient conditions, increased temperature, elevated CO_2, and the combination of elevated CO_2 and increased temperature, respectively. Different letters indicate significantly different by Tukey HSD test. SD, standard deviation.
